# Drivers of Antibiotic Resistance Transmission in Low- and Middle-Income Countries from a “One Health” Perspective—A Review

**DOI:** 10.3390/antibiotics9070372

**Published:** 2020-07-01

**Authors:** Katia Iskandar, Laurent Molinier, Souheil Hallit, Massimo Sartelli, Fausto Catena, Federico Coccolini, Timothy Craig Hardcastle, Christine Roques, Pascale Salameh

**Affiliations:** 1 Department of Mathématiques Informatique et Télécommunications, Université Toulouse III, Paul Sabatier, INSERM, UMR 1027, F-31000 Toulouse, France; 2 INSPECT-LB: Institut National de Santé Publique, d’Épidémiologie Clinique et de Toxicologie-Liban, Beirut 6573-14, Lebanon; souheilhallit@hotmail.com (S.H.); pascalesalameh1@hotmail.com (P.S.); 3 Faculty of Pharmacy, Lebanese University, Beirut 1106, Lebanon; 4 Department of Medical Information, Centre Hospitalier Universitaire, INSERM, UMR 1027, Université Paul Sabatier Toulouse III, F-31000 Toulouse, France; molinier.l@chu-toulouse.fr; 5 Faculty of Medicine and Medical Sciences, Holy Spirit University of Kaslik (USEK), Jounieh P.O. Box 446, Lebanon; 6 Department of surgery, University of Macerata, 62100 Macerata, Italy; massimosartelli@gmail.com; 7 Department of Emergency Surgery, Parma Maggiore Hospital, 43126 Parma, Italy; faustocatena@gmail.com; 8 Department of General, Emergency and Trauma Surgery, Cisanello University Hospital, 56100 Pisa, Italy; federico.coccolini@gmail.com; 9 Department of Trauma service, Inkosi Albert Luthuli Central Hospital, Durban 4091, South Africa; hardcastle@ukzn.ac.za; 10 Department of Surgery, Nelson Mandela School of Clinical Medicine, University of KwaZulu-Natal, Congela, Durban 4041, South Africa; 11 Departement of Bioprocédés et Systèmes Microbiens, Laboratoire de Génie Chimique, Université Paul Sabatier Toulouse III, UMR 5503, 31330 Toulouse, France; roques-ceschin.c@chu-toulouse.fr; 12 Department of Bactériologie-Hygiène, Centre Hospitalier Universitaire, Hôpital Purpan, 31330 Toulouse, France; 13 Faculty of Public Health, Lebanese University, Beirut 1103, Lebanon

**Keywords:** antibiotic resistance, one health, Low- and Middle-income countries

## Abstract

Antibiotic resistance is an ecosystem problem threatening the interrelated human-animal-environment health under the “One Health” framework. Resistant bacteria arising in one geographical area can spread via cross-reservoir transmission to other areas worldwide either by direct exposure or through the food chain and the environment. Drivers of antibiotic resistance are complex and multi-sectoral particularly in Lower- and Middle-income countries. These include inappropriate socio-ecological behaviors; poverty; overcrowding; lack of surveillance systems; food supply chain safety issues; highly contaminated waste effluents; and loose rules and regulations. In order to examine the drivers of antibiotic resistance from a “one health” perspective, a literature review was conducted on three databases including PubMed, Medline and Google Scholar. A total of 485 studies of potential relevance were selected, out of which 182 were included in this review. Results have shown that the aforementioned market failures are the leading cause for the negative externality of antibiotic resistance that extends in scope from the individual to the global ecosystem. Incremental and sustainable global actions can make the change, however, the problem will continue to prevail if governments do not prioritize the “One health” approach and if individual’s accountability is still denied in a world struggling with profound socio-economic problems.

## 1. Background

Antibiotic resistance is a multifaceted ecosystem problem that threatens the interdependent humans, animals and environmental health [1,2] linked together under the “One Health” framework. Resistant bacteria arising in one geographical area can spread via cross-reservoir transmission to other areas worldwide either by direct exposure or through the food chain and the environment [3,4,5] In this context, the drivers of resistance are complex, multifaceted and multi-sectoral. Humans and animals share not only the same environment but also common infectious diseases [6] that may have initially originated in animals [3,7]. The cross reservoir transmission from animal to humans potentially occur via animal contact [8,9] or from food sources [10,11,12] while transmissions from the environment to humans may occur via vegetable consumption [13] or water [14]. Wherever antibiotics are used, there are reservoirs of resistant genes that have the propensity to spread via ecological niches through complex “One Health” interactions between humans in different settings (i.e. community and hospital) and via animals (i.e. domestic or wildlife) and the environment [2]. The link between antibiotic uses and resistance may seem a clear cut, nevertheless, a causal relationship is not well established [15]. Drivers of resistance may be related to the dramatic changes in food consumption due to increased population density and subsequent enhanced needs for faster growth in the agricultural industry, the spread of resistance through international trade and travel, in addition to the global problems of sanitation, water contamination, sewage, manure run offs and waste including pharmaceutical industry waste and hospital waste. As example, the MCR-1 (mobilized colistin resistance-1) gene was initially detected in pigs in China in 2014 [16] and has subsequently spread to 50 other countries [17]. The New Delhi metallo-beta-lactamase 1 (NDM-1) that has originally emerged in India in 2008, has been detected later in the United Kingdom (UK) [18] as a result of medical tourism and became spread in the environment in Bangladesh in 2010 [19] and thereafter it was identified in the Arctic soil of the Norwegian archipelago potentially through migratory birds [20]. The magnitude and impact of these issues may vary from country to country and even per region in the same country particularly in Low- and Middle-income countries (LMICs). These regions also share some common global hurdles with high-income countries despite rigid policies and procedures [3,21,22]. The review examines the drivers of antibiotic resistance transmission in developing countries and the proposed strategies to mitigate this public health threat from a “One Health” perspective.

## 2. Method of Literature Search

Search methods for the identification of relevant studies was conducted on 24, January 2019, using the below three electronic databases:

- Ovid MEDLINE(R) Epub Ahead of Print, In-Process & Other Non-Indexed Citations, Ovid MEDLINE(R) Daily and Ovid MEDLINE(R) 1946 to Present
- PubMed http://pubmed.gov
- Google Scholar

The search strategy principle was based on dividing the topic into three concepts: (1) Antibiotic resistance and (2) One health and (3) Low-and Middle income countries.

All searches were limited to English language with no restriction on publication date to ensure that search results include all published articles pertained to the topic.

Ovid Medline was first searched to identify all the possible MeSH terms with their corresponding keyword equivalences to increase sensitivity of the search strategy. This technique utilized the many search options available for Ovid Medline such as Boolean operators, truncation, adjacency searching. 

The search strategy combined the three concepts as follows: one health.mp. OR exp. One Health/ec., lj., st., td. (Economics, Legislation & Jurisprudence, Standards, Trends) AND exp. drug resistance, bacterial/ or exp. drug resistance, multiple, bacterial/ OR ((antimicrobial or antibacterial or Microbial* or anti-microbial or antibiotic*) adj2 (resistant*)).mp. (mp. = title, abstract, original title, name of substance word, subject heading word, floating sub-heading word, keyword heading word, organism supplementary concept word, protocol supplementary concept word, rare disease supplementary concept word, unique identifier, synonyms) AND exp. developing countries/or third world.mp. or under-developed.mp. or developing nation.mp. or less developed nation.mp. or least developed country.mp. or least developed countries.mp. or least developed nation.mp. 

After finalizing the Medline strategy, the search terms were appropriately adapted into the other two databases. The obtained results were screened and studies were excluded if there primary objective was not solely the drivers of antibiotic resistance from One health perspectives. 

**Figure 1:**
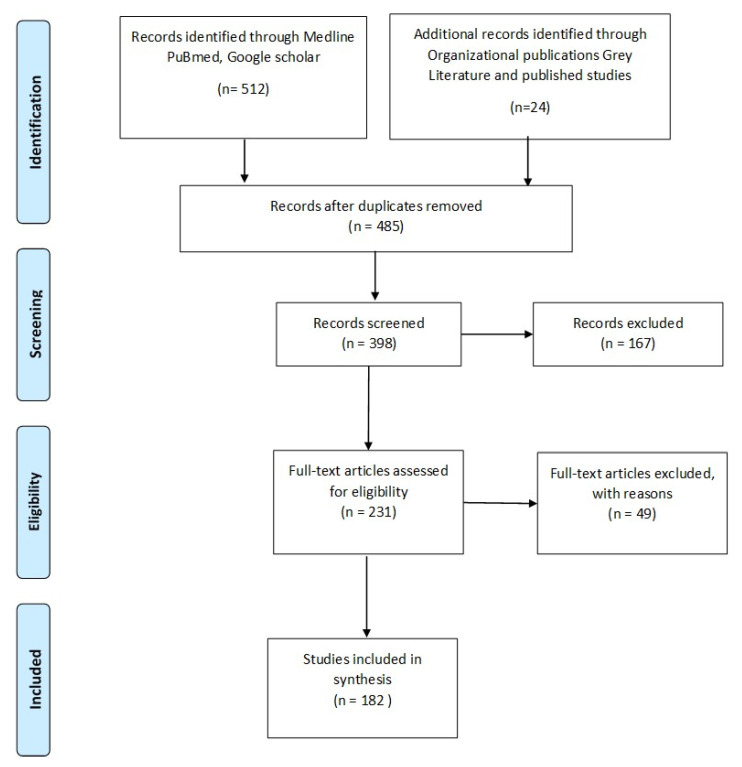
PRISMA flow diagram of the search.

Further search included articles through organizational publications and ‘grey’ literature that is, World bank, Centers for Disease Control and Prevention, World Health Organization and European center of disease prevention and control. Further studies were identified by hand search and by examining the reference lists of all included articles. A total of 485 studies of potential relevance were selected, out of which 182 were included in this review. (Figure 1- PRISMA flow diagram of the search).

## 3. The Environmental Resistome

**Figure 2:**
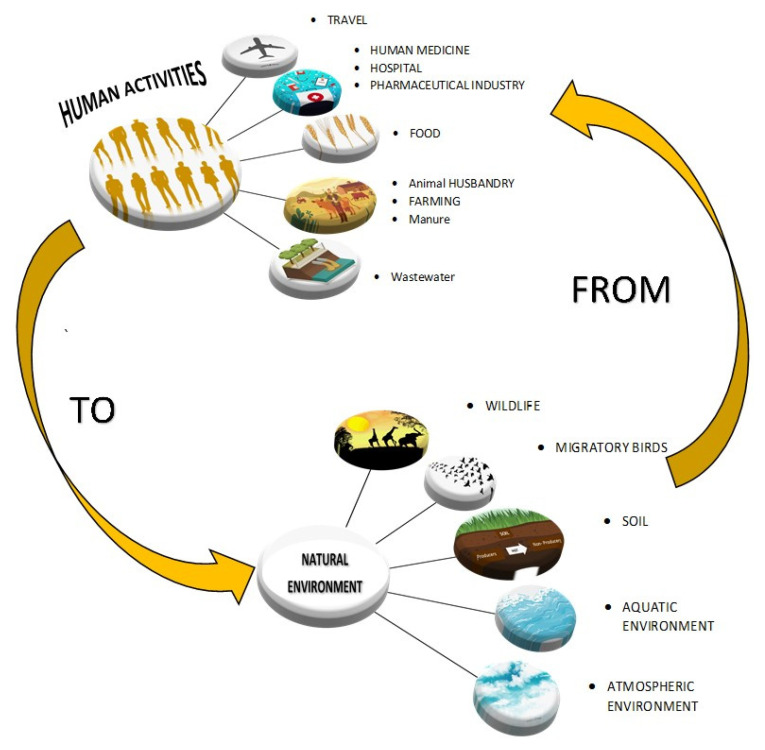
The environmental resistome.

The environmental resistome provides a clear understanding of the origins of antibiotic resistance. Compiling evidence has shown that the environment is at the same time the source and the reservoir for resistant genes [23,24,25]. The genetic and functional diversity in the resistome is the reflection of the evolutionary process of billions of years of bacterial co-existence with organic and inorganic compounds [25]. Resistant determinants found in environmental bacteria are genetically and mechanistically diverse [25,26]. These elements were long encoded in the bacteria genomes and has evolved in response to their exposure to organic and inorganic toxic substances [25] It is the continuous attempt to indiscriminately sterilize the environment through the use of antimicrobials agents, heavy metals and disinfectants [27,28] that has mainly contributed to the disruption of the ecosystem. The selective pressure and mobilization of resistance elements has led to the emergence and the flow of resistant genes from the environment to the pathogens [25,27]. Once mobilized and transferred in the pool of humans and animals, they provided significant opportunities for exchange and dissemination of resistance [28]. The resistome has both intrinsic and acquired resistant mechanisms. Intrinsic resistance are fixed in the core genetic make-up of the bacteria. They are chromosome encoded and include antibiotic inactivating enzymes, efflux pumps, permeability barriers and up-regulation of genomic mutation [29,30]. Intrinsic resistance may only be clinically relevant in immunocompromised patients since normal commensal flora or environmental bacteria have the potential to become opportunistic pathogens in these cases [31]. While, acquired resistance occur through the horizontal gene transfer (HGT) of resistant elements from other species [25] and genera and includes plasmid-encoded specific efflux pumps, altered targets and drug inactivation [32]. These mechanisms are clinically relevant and considered a serious threat to human health because of a change in the context of the resistance determinants from chromosomal to HGT mediated resulting in their enhanced expression and dissemination [26,33]. This has been demonstrated as a major concern worldwide and particularly in Lower- and Middle-Income Countries (LMICs) [34,35,36]. Nevertheless, with the increased evidence that antibiotic resistance origins and subsequent evolution reside in environmental pathogens, exploring and understanding the associated molecular mechanisms and genetic mobilization modalities can potentially provide new lead for novel drug discovery [25]. (Figure 2-The environmental resistome).

## 4. Drivers of Antibiotic Resistance Transmission

### 4.1. Socio-Economic Factors and Related Socio-Ecological Behaviors 

In a developing word with scarce resources, poor sanitation and food safety issues, loose rules and regulations, overcrowding and poverty, the economic drivers of inappropriate socio-ecological behaviors are one of the leading cause of antibiotic resistance (ABR) [37,38,39]. A study conducted in Madagascar have shown that the high socio-economic status, housing quality, access to clean water and occupational status have lower risk of colonization with ESBL-producing Enterobacteriaceae [40]. While other studies reported households sharing common habitats with livestock or poultry in rural areas in India [41] and Bangladesh respectively [42]. The combination of poverty, poor regulations and lack of knowledge is a recipe for detrimental behaviors and practices. As example, raising backyard poultry in rural areas in Bangladesh brings a good return-on-investment, however these practices are associated with low or inexistent biosecurity measures [43]. Poor sanitation and hygiene was reported in different studies after slaughtering food-animals and butchering poultry [43,44,45]. Slaughters’ and butcheries waste are released in the environment (i.e. open land and municipality water), allowing domestic and wildlife animals to scavenge on these leftovers. Untreated animal waste is used for a variety of purposes to generate income among which the application of animal manure as fertilizers [37]. Other socio-ecological behaviors include sharing surface water between humans and animals. Humans’ uses water for cleaning, fishing and bathing while animals use the same water for drinking, grazing and defecating [46]. The growing demand for animal protein [37,38,39] mainly in Asia and Africa and to a lesser extent in South America has triggered the routine use of antimicrobials to maintain animal’s health and meet the global transition for high-protein diet (i.e. meat, poultry, egg, fish), urbanization and the need to intensify animal production [47]. The poorly regulated food supply chain mainly in Africa and Southeast Asia [48] has led to the spread of resistant bacteria particularly foodborne pathogens. In Ethiopia, antibiotics are being added directly to raw milk in an attempt for the vendors of extending shelf-life of their products [49]. Food supply chain safety is a major issue especially in the absence of data to track the sources of contamination, in addition to poor or absence of regulations in the agricultural sector. Studies conducted in different countries namely Ghana [50], Nigeria [51], Senegal [52], Kenya [53] and Tanzania [54] showed high antimicrobial residues in eggs and meat compared with Europe [55], a highly controlled and regulated area. A qualitative research examined Cambodian workers [56] involved in food-chain showed that the main drivers of inadequate farmers practices are related to poor control systems, unlimited access to antibiotics and strong belief about the necessity of antibiotics for animal raising due to lack of knowledge about the deleterious effects of these practices. (Table 1- Socio-economic drivers and inadequate socio-ecological behaviors in LMICs).

### 4.2. Antibiotic Uses in Human Medicine

Antibiotics are one of the most extensively prescribed medications in human medicine [57,58]. Studies have shown that these drugs are being misused, overused or underused in low-, middle- and high-income countries [5,57,58,59,60,61]. Antibiotic consumption is thought to be the major driver of ABR [62,63,64,65]. Global estimates indicate that only 50% of the global antibiotic consumption is appropriately justified [66]. In the US, the Center for Disease Control and Prevention (CDC) published the results of data analysis from the National Ambulatory Medical Care Survey (NAMCS) and the National Hospital Ambulatory Medical Care Survey (NHAMCS) that showed, that only 30% of 154 million antibiotic prescriptions written yearly in physician’s offices and emergency departments, are considered unnecessary. The same report indicated that 44% of outpatient antibiotic prescriptions are intended to inappropriately treat acute respiratory conditions (e.g. viral upper respiratory tract infections, bronchitis, asthma, allergies and influenza) [47]. The high percentage of unnecessary antibiotic prescriptions is a common finding across the literature [67,68,69,70,71]. In Europe, antibiotic use is the highest in hospital settings and constitutes 30%–40% of inpatient medication prescriptions [72]. In Canada, consumption of antibiotic in the hospital is lower compared to the community (30% versus 70% respectively), however, the estimated inappropriate prescribing is higher in the hospital setting [59]. This means that inappropriate antibiotic use is a global issue that indiscriminately affects LMICs as well as high income countries (HICs). One of the reasons is because the incentives for antibiotic misuse and abuse outweigh the incentives for the right use of these medications [66]. In the hospital setting, the leading cause for antibiotic misuse is not clear. However, the time pressure constraints especially in the emergency room, leadership and governance in the applicability of related policies and procedures, the absence of rapid diagnostic tests to guide prescribing [73], the eligibility of antibiotic prophylaxis in invasive procedures, chemotherapy and intensive care units pitfalls, in addition to the challenges of treating nosocomial infections [5,68,69,70,71] all impact ABR. While in the community setting, antibiotic abuse may be linked to the physician prescribing behavior [74], to the diagnostic uncertainty [75] and to the patient behavior (i.e., failing to finish the full course of antibiotic therapy or demands for unnecessary treatments due to fear or lack of understanding of the risks), to stockpiling leftover for future uses and auto-prescription and to the level of public awareness about antibiotic uses and the concept of antibiotic resistance. The consumer may also be driven by the low cost of medications, the availability of antibiotics over-the-counter in some countries [74,76], the lack of awareness about the link between irrational antibiotic uses and ABR [66]. In LMICs, potentially unreliable microbiology and laboratory results in some settings may be the leading cause for the increased use of broad spectrum antibiotics and sometimes for unjustified combinations or length of treatment. In these countries, inequality of access to medications, due to poverty may drive the patient to underuse antibiotics or to unknowingly choose counterfeit medications due to lower price [5,68,69,77]. In HICs, estimates show that despite the established guidelines for justified uses of antibiotics, evidence of lower rates of antibiotic prescription and an overall reduction in prescriptions over the past decade, has only resulted in a modest reduction in antibiotic resistance [78,79]. This means that the causal association is much more complex [80]. The selection pressure and antibiotic resistance causal relationship should be addressed carefully taking into consideration multiple confounding factors specific to each drug and to each microorganism [5,80] including pathogen-pathogen and pathogen-host interactions, mutations, cross-resistance, mechanisms of resistance and cross-reservoirs drivers.

### 4.3. Counterfeit Antibiotics

The influx of counterfeit antibiotics into the global pharmaceutical market is estimated at 5% [81]. This serious health threat is a worldwide problem sparing no borders and no country, especially in developing countries and to a lesser extent in developed countries [81,82,83]. The majority of these products originating from south-East-Asia and Africa, are destined mainly to emerging countries including South-East –Asia, sub-Saharan Africa, Europe and North America81. Counterfeit antibiotics are a type of substandard drugs. In the absence of an international consensus for the definition of counterfeit [84], the World Health Organization (WHO) defines the term as “drugs that are deliberately and fraudulently mislabeled with respect to identity and/or source” [85]. Counterfeit may contain the correct or the wrong ingredients and either a sub-therapeutic dose [81,83,86] to no active ingredient at all [82,87] or fake packaging leading primarily to the selection of antibiotic resistance, uses of broad spectrum antibiotics, treatment failure and subsequently morbidity and mortality [82,83,88,89]. Half of the reported counterfeit antibiotics are beta-lactams followed by quinolones, macrolides and to a lesser extent lincosamides among other antibiotics. An estimated 77% of these drugs are intended for oral administration (i.e. tablets, syrup and capsules), 17% are formulated for parenteral administration and the remaining are either eye drops or ointments dosage forms [81]. These products are used to treat people with serious infections among which children, pregnant women and frail elderly people. Although it is a worldwide commonly occurring problem, it is still not eradicated and continues to exert a devastating negative impact mainly because of the lack of regulations, law-enforcement and poverty. 

### 4.4. Non-Prescription Antibiotics

At an increasingly unprecedented level on an international basis, antibiotics are becoming more and more available over-the–counter or via unregulated supply chains [89,90]. Access to antibiotics can be as easy as a few clicks away using the internet, where these drugs can potentially be ordered and delivered to the consumer anywhere in the world through illegal online vendors [67,91]. Recent evidence has shown that this problem is seen in developing as well as in some developed countries [92,93] while many others legally prohibit the sales of antibiotics over-the-counter [94,95,96,97]. This results from weak law enforcement or even the absence of policies and regulations [94,95,96,97,98,99,100]. It is once again the poor law enforcement and the lack of policies and regulations that has led to escalating sales reaching a critical level of up to 50% of total antibiotics sales worldwide [101]. Estimated related figures range between 20% to 30% in Southern and Eastern Europe (i.e. Spain and Portugal) [102] and up to 100% in parts of Africa [103]. This public health issue is mainly encountered in the community settings since the retail pharmacies are the patient’s first point of contact with a healthcare provider [104,105]. A systematic review and meta-analysis by Auta et al. [94] showed that non-prescription antibiotics are mainly supplied upon patient request or to a lesser extent according to the pharmacist’s advice. According to the review, the most frequently treated infections were urinary tract infections and upper-respiratory tract infections while the most consumed antibiotics in these cases were classified as high priority drugs by the WHO (i.e. Fluoroquinolones and Penicillins) [106]. In developing countries mainly Africa, the community is providing different unauthorized services like consulting, diagnosing, prescribing and dispensing medications [107,108]. These illegal practices can lead to the misuse and abuse of antibiotics. This in turn can increase the selection pressure and lead to antibiotic resistance. Nevertheless, in some developed countries, non-prescription sales of antibiotics by a pharmacist are legally supported. In the United Kingdom, retail pharmacist can sell azithromycin to patients with positive chlamydia test results, while in Canada and New Zealand a designated pharmacist is given the authority to prescribe and dispense antibiotics according to strict validated conditions [104]. Managing non-prescription antibiotic sales is possible and multiple interventions have been proposed among which law enforcement and sales restrictions, pharmacist training and empowerment. These measures can potentially lead to a decrease in the use of antibiotics over-the-counter [109,110,111] and reduce the selection pressure but not necessarily reverse antibiotic resistance [112].

### 4.5. Antibiotic Uses in Animal Health and the Agricultural Sector

Antibiotics are an integral part of the agricultural sector intended to enhance food production in order to meet the increased needs for human consumption. Data show that among different countries currently using veterinary antibiotics, five countries are projected to have the greatest percentage increases by 2030 namely Myanmar and Indonesia followed by Nigeria and Peru and to a slightly lesser extent Vietnam [39]. Antibiotics are extensively used to compensate poor sanitation, to prevent diseases in herd/flocks, to treat bacterial infections and to promote animal growth in the distinct agricultural sectors [113] (i.e. antibiotics are intended for prophylactic, metaphylactic and treatment purposes). The magnitude and contribution of cross reservoir resistance transmission from animal to humans is difficult to quantify and track mainly because of globalized food trade [114] and differences in the management of antibiotic uses in the agriculture industry. In livestock, these differences are mainly related to the husbandry styles, the routes of antibiotic administration to animals (i.e. orally by feed or water or by intramuscular or subcutaneous injection), distinct type of approved antibiotic per species, different doses and dosing regimens of antibiotics, variable duration of antibiotic use, timing of administration and drug withdrawal. All of these factors may vary from country to country [113,115]. Substantial evidence supports the link between antibiotic resistance in livestock and the emergence of bacterial resistance in humans [15,77,116,117]. Enteric bacterial isolates, detected in food-producing animals and meat and in particular in poultry and chicken meat isolates are commonly resistant to antibiotics critically important to human medicine like third-generation cephalosporins and quinolones in addition to ampicillin, tetracyclines and co-trimoxazole [118]. This is true in LMICs as well with poultry and pork farming the major role players as generators of ABR across a range of bacterial culprits [119,120,121,122]. An alarming concern, is the acquired colistin resistance in food-borne pathogens seen across several countries in animals and humans (i.e., associated with infection) [123]. This evidence supports the contributory effect of international trade and travel. The vast majority of antibiotics are intended for common use by both humans and animals, few are exclusively reserved for humans (i.e., carbapenems) and the remaining other few are limited to the veterinary sector mainly because they are toxic to humans. The most widely consumed antibiotic in animals is oxytetracycline, followed by norfloxacin, enrofloxacin, erythromycin, sulphonamides and co-trimoxazole [124]. Nevertheless, data related to the extent and patterns of antibiotic uses in food-producing animals especially in LMICs are limited [125]. In these countries, antibiotics are being used by farmers without veterinary supervision because of their low cost and availability over-the-counter [98]. While food is likely to be a major vehicle for transmission of resistance from animals to human additional ways of cross reservoir transmission are direct zoonotic transmission including from pet animals, risks related to close contact with animals in the meat production industry and environmental contamination resulting from farm manure-contaminated water runoff (e.g., excreted antimicrobials or their metabolites, residue concentrations of antimicrobials in edible tissues) [77,126,127,128]. Antibiotic resistance is also spread in the aquatic environment and fish gut as a result of the contamination with human and animal waste [118,129,130]. In LMICs, transmission of resistant genes to farm fish occur by using domestic farm and poultry waste as fish feed [131] or occasionally through bath treatments. Some studies have demonstrated similarity between acquired resistance genes and the associated mobile elements of fish and shellfish pathogens and those isolated from clinical bacterial isolates. This means that they may potentially share common origins and gives an idea on how these pathogens can be transferred from humans to fish [132]. Treatment of ailing fish with antibiotics used for human medicine and then dumping these treatments directly in to the water or via fish food is one of the leading cause of bacterial resistance in the aquatic environment. An estimated 80% of antibiotics used in aquaculture are found in the aquatic and terrestrial environment. This can contribute to the selection pressure of bacteria and alteration of the biodiversity of the aquatic environment through bacterial mutations and transfer of mobile genetic elements containing different resistant determinants. The commonality of the mobilome between aquatic and terrestrial bacteria may have originated from human and animal pathogens [133]. Studies have shown that aquatic bacteria resistant to antibiotics like fluoroquinolones, tetracyclines, flofenicol has been isolated among which Escherichia coli, a pathogen found in humans [134] Several genetic elements and determinants for tetracyclines, quinolones and β-lactamases may have originated in aquatic bacteria are shared between human pathogens, aquatic bacteria and fish pathogens. However, there are no conclusive evidence of a human disease-causing agent acquiring resistance from aquaculture origins [135].

### 4.6. Other Drivers of Resistance

Other drivers of resistance within the “One Health” framework include Heavy metals, biocides and wastewater associated with the co-selection and dissemination of resistance in environmental bacteria [136]. Heavy metals are found in the soil from agriculture and mining origins and as trace elements of antimicrobial growth promoters (AGPs) used in livestock production. Biocides are frequently encountered within the food and agriculture industry intended for use as animal food preservatives and within the healthcare settings as disinfectants and decontaminants. These are associated with the dissemination of AMR in environmental bacteria due to decreased susceptibility of bacteria in soil [137]. Biocides and antimicrobial agents can share common target sites [138] and can be located closely together in mobile units [139]. Many genes encoding resistance to these agents has been linked to antibiotic resistant genes in single genetic elements leading to cross and co-selection for resistant pathogens [138,140]. Wastewater is the direct result of anthropogenic activities [141]. High amounts of antibiotics (i.e., human and animal sources) are released in the environment leading to the selection of ABR and the spread of antibiotic resistant genes (ARGs). Untreated wastewater discharge from pharmaceutical companies and hospitals [77,142,143], manure runoffs, slaughterers markets liquid waste, farms harbors and untreated livestock waste, constructed wetlands sediments, broiler feedlots and fishponds are the main sources of aquatic ecosystem disruption. These are considered ideal sites for the occurrence and the spread of (ARGs) that accumulates and transfer clinical pathogens through HGT to the entire ecosystem [141,144].

## 5. The Economics of One Health 

Tackling antibiotic resistance from the “One Health” perspective is embraced by the WHO/FAO/OIE Tripartite, the Declaration from the 2016 high-level meeting on antimicrobial resistance at the United Nations General Assembly and is supported by the World Bank [1,145,146,147,148,149]. “One Health” models are thought to engender broad effectiveness and efficiency outcomes generating savings in operating costs. These models are based on building veterinary/human public-health capacity and enhancing awareness in order to reach effective global governance. Capitalizing on these capacities especially in low-income countries will eliminate the leading causes of antibiotic resistance and infections pandemic. The Canadian Science center for Human and Animal Health is the first organization worldwide to house in one facility the laboratory for human and animal diseases research. Extrapolating data from this report in addition to validated assumptions for a panel of experts has led to the conviction that implementing the “One Health” surveillance approach in the 139 World Bank client countries [150] (i.e. Classified as LMICs as of 2008) can generate substantial amount of savings ranging from US $184 million per year in low disease-prevalence scenario to US $ 506 million per year in high-disease prevalence scenario excluding the savings generated from planning, communication, education, natural resource benefits, training or research [2]. In turn, the projected added value of “One Health” in action for tackling ABR in terms of cost and cost containment is projected to require a cumulative investment of US $0.1 trillion at a steady pace between 2017 and 2030 as allocated costs in order to generate lower healthcare expenditures yearly by as much as US $0.22 trillion in 2030 if the low-AMR scenario is avoided and by as much as US $0.7 trillion if the high-AMR scenario is avoided [2,66,150]. Allocation of funds and investments in the human and animal health sector differ in terms of prevention of zoonotic disease (i.e. surveillance, diagnostics and biosecurity) and control measures (vaccination and hygiene programs, investigation and rapid response in the human health sector and compensation and culling in the animal health sector). Budgets allocated in human health sector range from 70% on control and 30% on prevention while the opposite prevail in animal health sector where 50%–70% is allocated for prevention and 30%–50% for control measures [150].

## 6. Surveillance of Antibiotic Consumption in Humans and Animals

There are multiple indicators for weak governance associated with inappropriate use of antibiotics in humans and animals. As data on the patterns and amount of antibiotic consumption is limited, the foundation to fill this gap is setting a global surveillance system [60,77,135,151,152]. In this context, the WHO methodology for a global antibiotic surveillance program provides a standardized data collection and data sharing nationally and internationally and allows access to important information in the fight against AMR [151,153]. This approach is also adopted by the European surveillance of antimicrobial consumption Network (ESAC-Net) and the WHO Regional Office for Europe. Data findings advise policies, regulations and interventions aiming at optimizing the uses of antibiotics. This project is part of the WHO Global Action Plan (GAP) [153] that will be included in the Global Antimicrobial Surveillance System (GLASS) platform. In 2018, the WHO [151] report on surveillance of antibiotic consumption in 65 countries showed the variability in the amount and patterns of antibiotic use on regional, national and global levels. Strengthening the surveillance of antibiotic consumption is based on five dimensions: (1) Integration of surveillance in humans, food-producing animals, pets, plants and aquaculture and animal; (2) Coherence using standardized methods for reporting and data collection; (3) breadth by including a wider range of antibiotics and biocides; (4) Innovation by using new tools and technologies to improve surveillance; (5) cooperation between relevant stakeholders. The costs allocated to implement and maintain a surveillance system depend on national data requirements, priorities and capacity [154]. It varies according to the availability of advanced and well-established surveillance system, whether the surveillance of antibiotics is embedded in a wider healthcare infrastructure or not and on the degree of microbiological laboratory reliability. Unfortunately, many LMICs lack capacity to establish this system and monitor antibiotic consumption. This is why funding is essential to provide financial and technical support for building laboratories and enhancing capabilities. The sources of funding can be from governmental and non-governmental entities. As example, the fund from the US government via the Global Health Security Agenda (GHSA) and from the UK Government with its announcement of the (375 million USD) Fleming Fund [77] to support the implementation of this system in LMICs. While surveillance of antibiotic consumption is fundamental, a massive global public awareness campaign is as important to enhance knowledge about AMR in general and antibiotic uses and resistance in particular. A shift toward a positive behavior change tailored to local and regional norms can be done via social media, broadcast advertisement, trainings patients, farmers, healthcare professionals, veterinarians about antibiotic uses and antibiotic resistance and via tackling mainly the youth. These programs may be supported by the United Nations (UN) assembly, international campaign developers, industry experts and non-governmental organizations. The costs of running a sustained global public awareness campaign varies depending on its nature and scope. Costs estimates may between US $ 40 and $100 million per year [5,66,155].

## 7. Surveillance of AMR in Humans and Animals 

Surveillance systems are the foundation for a better understanding of the epidemiology of antibiotic resistance and the key for tackling this public health threat [66,156]. According to the WHO, surveillance should be an essential part of every national antibiotic resistance action plan [66,149]. Without adequate surveillance, the majority of efforts to contain resistance will be challenging and often difficult. Surveillance data may serve on local, national and international level to improve public health, inform health policies, develop evidence-based policies, trigger responses to health emergencies, provide early warnings of emerging threats and identify long–term resistance trends [77]. AMR surveillance in humans has received more focused attention compared with AMR in animals despite the compiling evidence that the spread resistance has a zoonotic origin [125]. Currently the only country that has publicly available surveillance data on AMR in animals is Colombia [125]. The raising rates of resistance in animals is projected to have detrimental health consequences for humans and animals [39]. Since two decades ago, the proportion of antimicrobials resistance has at least doubled in chickens and in pigs [39]. China and India represent the largest hotspots while Brazil and Kenya are new emerging hotspots van. Mapping the global trends of resistance in humans, animals and the environment framework is a highly challenging project. Data are scare in LMICs in the presence of multitude of hotspots and associated barriers [39]. However, the number of studies and surveys has increased in the past decade. Surveillance should encompass two major areas in addition to the magnitude of antibiotic consumption—(1) types, rates and extent of antibiotic-resistant bacteria and (2) mechanistic basis of antibiotic resistance [57,66,77,157]. In order to achieve these goals, a global coordinated and standardized action should take place with the collaboration of the WHO, Food and Agriculture Organization (FAO), World Organization of Animal Health (OIE) [66,77,149,158], regional bodies, philanthropic organizations and governments. These initiatives (i.e. GLASS; European Antimicrobial Resistance Surveillance Network (EARS-Net), Central Asian and Eastern European Surveillance of Antimicrobial Resistance (CAESAR), Healthcare-associated Infections Surveillance Network (*HAI*-*Net*) can increase and strengthen international cooperation and support capacity building especially in LMICs. Nevertheless, the challenges are enormous due to weak laboratory and communications infrastructure, lack of financial and technical expertise [125,159]. In order to do that, an assessment should be conducted on the readiness to participate in national and regional AMR surveillance. Current surveillance capabilities are variable across the world. Europe and the USA have the best surveillance coverage while the Sub-Saharan Africa and South and Southeast Asia have the least developed66. In LMICs, the challenges are enormous due to weak laboratory and communications infrastructure, lack of trained and qualified staff and higher incidence of counterfeit antibiotics [160,161]. Questions may be raised about the feasibility and the affordability of implementing a global surveillance system. A six-month surveillance program in Ghana demonstrated the feasibility of the project [127]. Concerning the affordability, establishing a reliable estimate on the costs of implementing a comprehensive global surveillance system in humans, animals and in the environment is very challenging. Kenya, one of the countries participating in the East Africa Public Health Laboratory Network (EAPHLN), is constructing a national AMR surveillance network at an estimated cost of US $160,000. This cost can be extrapolated to estimate the global allocated budget for implementing such program in other similar countries. The World Bank has recommended an estimated US $ 9 billion per year for the containment of AMR. About half of which is for building core veterinary and human public–health capacity in LMICs with the collaboration of different stakeholders including the WHO [66]. A figure that may sound very expensive but looking at the priceless long-term benefits and the countless cost of inaction will shift the paradigm from cost to high level of return on investment [162].

## 8. Adaptation of Preventive Measures Strategies

Universal Health Coverage (UHC) reform is one of the strategies to combat AMR, currently adapted in many countries. The UHC can build system-governance and coordination capacities that are highly relevant for tackling AMR crisis. Under this model, governments can take informed decisions and allocate wisely health investments in areas such as Infection Prevention and Control (IPC) and vaccines [66]. 

### 8.1. Antibiotic Stewardship Programs

UHC models can accelerate gains in AMR through improved health information, effective prevention of infections and evidence-based antibiotic stewardship programs [77,163,164]. Under UHC model, regulatory capacities such as hospital accreditation, can be enhanced. The UHC model strengthens antibiotic stewardship and reinforces the uses of standard antibiotic regimens for the treatment of infections thus optimization of antibiotic use. This program has been implemented in some countries with impressive results [53] leading to a reduction in the uses of antibiotics especially broad-spectrum antibiotics in addition to the decrease in healthcare costs and the improvement of patient outcomes and AMR containment [67,165]. Similar programs in South Africa, a lower-middle income country, in both the private and public hospital sectors, have shown reductions in inappropriate antibiotic use and that the correct antimicrobial choice can be determined through effective surveillance [166,167,168,169,170].

### 8.2. Vaccines

One health interventions vaccinations programs (i.e. veterinary vaccines and human vaccines) are cost-effective approach for the prevention of endemic and neglected zoonoses in animals [171] and multiple communicable diseases in humans. Vaccines can decrease selection pressure [66,77,155,172]. However, the lack of knowledge, myths and poverty has contributed to the low use of these medications especially in LMICs [173]. Currently, the global market share of vaccines of the global pharmaceutical market in only 3% [174]. A study conducted in the US in 2011 showed that pneumococcal vaccine led to 64% and 45% reduction in antibiotic–resistant pneumococcal infections respectively among children and adults over 64 years [175]. Universal coverage is expected to prevent 11.4 million days of antibiotic use per year in children less than 5 years of age and an estimated 47% reduction in antibiotics used to treat these pneumococcal infections [176]. According to the CDC, there are no licensed vaccines for any of the bacterial species considered an urgent AMR threat [165]. The global innovation fund supporting basic and non-commercial research in the pharmaceutical domains among which vaccines is estimated to be up to US $2 billion over 5 years [177]. Veterinary vaccines should be readily available to farmers [38] who should be educated about the uses and benefits. However, available vaccines do not cover all type of animals or some types of low-value fish raised or farmed in some regions in LMICs [38].

### 8.3. Rapid Diagnostic Tests 

Today, antibiotics are prescribed either empirically or “just in case.” They are very rarely based on definitive diagnosis because the laboratory tests still rely on the processes developed in the 1860s. The lapse of time exceeding 36 hours fosters diagnostic uncertainty and leads to over-prescribing of antibiotics to eliminate risk perceptions. Promoting new rapid diagnostic tests can be considered critical for a drastic shift in the practice [57,73,178] and a substantial decrease in unnecessary prescribing. However, it may not represent an incentive to pharmaceutical companies to invest in R&D especially generic companies because of the financial implication on sales may be huge. In LMICs, where affordability and easy access to diagnostics are the main barriers, using a Diagnostic Market Stimulus (DMS) is estimated at an average yearly cost of US $0.5 to $1 billion based on the cost of current available diagnostic tests. Recent developments include the access to and use of biomarkers, such as procalcitonin, to reduce antibiotic duration and for early cessation of therapy [178].

### 8.4. Pharmaceutical Waste

Another intervention within the scope of antibiotic use is related to the substantial amount of waste generated from antimicrobial activity through the antibiotic supply chain that can be prevented from reaching the environment and increasing the cross-reservoir transmission of resistance. The estimated costs for the preventive action based on a rough 30,000 to 70,000 tons of waste is around US $180 million annually that is, US $0.5 /kilogram of Active Pharmaceutical ingredients (APIS) produced. This quantity represents 10–20% of the 250,000 tons of global antibiotic consumption per year [77,178]. 

## 9. Highlights

The challenges in LMICs are complex and multi-sectoral. The core issues are related to socio-economic limitations and inadequate socio-ecological behaviors. Poverty, lack of education and training combined with loose rules and regulations are the recipe that fuels antibiotic resistance in regions known for their endemic resistance epidemiology. One health” is a public good that has the potential to mitigate the negative externality of antibiotic resistance. Although global actions had been undertaken, high level of support, allocated funds training and surveillance systems has been put in place, the problem still prevail because it is not considered a public health priority in many countries. It may be due to limited resources in failing economies, to war and conflicts, to epidemics and pandemics and to political issues in certain “no voice” communities. However, if the detrimental impact of antibiotic resistance has expanded onward from the individual to the entire globe and ecosystem, the fight should be shifted inward and tackle mainly the individual and their governments. Education, training and extensive research are the foundation for shifting the paradigm. Incremental and sustainable coordinated global action can make the change only if there is a shift in socio-ecological behaviors and acceptance of government’s and individual’s accountability is not still ignored or denied. 

## 10. Conclusions

In a world struggling with profound socio-economic problems, the implementation of preventive measures, surveillance and rigid controls is expensive but the end-results will yield a positive externality that will impact uniformly on global health, the economy and the ecosystem under a “One health” umbrella.

## Figures and Tables

**Table 1:** Socio-economic drivers and inadequate socio-ecological behaviors in LMICs.

Counterfeit antibiotics
Availability of antibiotics over-the-counter
Limited public awareness and knowledge about antibiotics and antibiotic resistance
Strong belief that adding antibiotics to animals feed and drinks is part of raising healthy animal
Lack of food safety measures and controls
Lack or inadequate food safety regulations
Unmonitored food supply chain
Added antibiotics directly to dairy products in order to extend their shelf life
Inappropriate amount of antibiotics used to grow livestock, poultry and aquatic animals
Need to intensify food-animal and aquaculture production to meet the populations accelerated growth demands
Increase demand on meat due to urbanization, high-protein diet
Overcrowding
Household sharing habitat with poultry and livestock
Shared surface waters by humans and animals
Poverty and related economic and political drivers
Poor sanitation and hygiene
Poor farming hygiene
Veterinary vaccines are unavailable to farmers (either due to poverty and/or ignorance)
Eating behaviors and preferences (i.e. raw or undercooked meat)
Absence of farm biosecurity and frameworks for training farmers
High levels of environmental contamination with antibiotic residues, heavy metals and biocides
Behaviors relating to the slaughter and processing of food-animals (e.g. modalities of animal waste disposal, uses as animals feed)
Irrigation with untreated wastewater due to water shortage and poverty
Untreated animal waste is used for a variety of purposes including their use as fertilizers
Poultry waste is commonly used for feeding of fish and shellfish in aquaculture
Untreated wastewater originating from pharmaceutical industries, hospitals markets, manure and sewage runoffs
Animal waste is often discarded on open land and then after consumed by domestic and wildlife animals
Liquid waste from markets, including blood, feces and wastewater is disposed into municipal drains through direct wash out
Surveillance systems of antibiotic consumption and epidemiology of ABR are emerging trends
The epidemiology of antimicrobial resistance (AMR) in animals are poorly documented
Lack or scarce evidence-base data on the magnitude and economic burden of AMR in humans
The need for strong laboratory capacity
Lack the financial capacity for establishing an adequate surveillance program

## Abbreviations

ABR	Antibiotic resistance
ARG	Antibiotic resistant genes
AGPs	Antimicrobial growth promoters
MCR-1	mobilized colistin resistance-1
NDM-1	New Delhi metallo-beta-lactamase 1
UK	United Kingdom
WHO	World health organization
US	United States of America
CDC	Center of Disease Control and Prevention
NAMCS	National Ambulatory Medical Care Survey
NHAMCS	National Hospital Ambulatory Medical Care Survey
LMICs	Low- and Middle-income countries
HICs	High income countries
FAO	Food and Agriculture Organization
OIE	World Organization for Animal Health
UN	united Nations
ESAC- Net	European surveillance of antimicrobial consumption Network (ESAC-Net)
GAP	Global Action Plan
GLASS	Global Antimicrobial Surveillance System
GHSA	Global Health Security Agenda
UHC	Universal Health Coverage
IPC	Infection prevention and control
DMS	Diagnostic Market Stimulus
APIs	Active Pharmaceutical ingredients
R&D	Research and development
